# Recreational versus self-medicated cannabis use among adolescents and young adults in Spain: associations with substance use and mental health

**DOI:** 10.1186/s42238-026-00480-2

**Published:** 2026-08-04

**Authors:** Roberto Secades-Villa, Sara Weidberg, Alba González-Roz, Layla Alemán-Moussa, Clara Iza-Fernández

**Affiliations:** Addictive Behaviors Research Group (GCA), Department of Psychology, University of Oviedo, Plaza Feijoo, Oviedo, 33003 Spain

**Keywords:** Cannabis, Adolescents, Young adults, Use motives, Substance-related problems, Mental health

## Abstract

**Background:**

Cannabis use is common among adolescents and young adults, yet motives, especially self-medication, remain poorly understood in non-legalized contexts. This study explored differences among recreational, self-medicated, and dual (recreational + self-medicated) cannabis users in sociodemographic, substance use, and mental health characteristics within a large Spanish sample of adolescents and young adults.

**Methods:**

Data were collected from 1,078 participants aged 16–30 who reported past-year cannabis use. Based on motives, users were categorized into three groups. Validated measures assessed cannabis use severity, concurrent substance involvement, nicotine dependence, alcohol-related consequences, and mental health. Bivariate analyses and multinomial logistic regressions were performed to identify predictors of group membership.

**Results:**

Dual users comprised most participants (53.2%) and showed the most severe profile, including higher cannabis use frequency, greater scores on the Cannabis Abuse Screening Test (CAST) and the Brief-Marijuana Consequences Questionnaire (B-MACQ), more tobacco co-use, and poorer mental health. Self-medicated users (8.3%) showed greater nicotine dependence, higher risk for cannabis use disorder, and higher scores on the Reality Distortion subscale of the Oviedo Schizotypy Assessment Questionnaire-Abbreviated (ESQUIZO-Q–A) than recreational users (38.5%). Multinomial logistic regressions identified cannabis-related problems and use frequency as the strongest predictors of dual or self-medicated use.

**Conclusions:**

Distinguishing cannabis use motives in adolescents and young adults is crucial, as dual users represent a highly vulnerable subgroup with substance-related and psychological risks. Prevention and intervention efforts should include motive-specific screening, address misconceptions about self-medication, and integrate mental health support, especially amid Spain’s evolving cannabis regulations.

**Supplementary Information:**

The online version contains supplementary material available at https://doi.org/10.1186/s42238-026-00480-2.

## Introduction

Cannabis is the most widely consumed illicit psychoactive substance worldwide and remains highly prevalent among adolescents and young adults (United Nations Office on Drugs and Crime 2023). In Spain, the lifetime prevalence of cannabis use among adolescents approaches 40% by age 18 according to national surveys (Observatorio Español de las Drogas y las Adicciones 2023).

Adolescence and young adulthood are periods of heightened sensitivity to substance-related harms. Neurodevelopmental processes during these years render individuals more vulnerable to the cognitive and psychiatric consequences of cannabis use (Debenham et al. 2021). Meta-analytic evidence indicates that heavy or frequent cannabis use is associated with small but significant deficits in attention, memory, and learning, although some effects attenuate with abstinence (Scott et al. 2018). Longitudinal research shows that cannabis use and cannabis use disorder (CUD) are associated with psychiatric comorbidity (Muñoz-Galán et al. 2023; Burke et al. 2024). Moreover, concurrent use of alcohol, tobacco and cannabis is common, and this pattern of polysubstance use is associated with greater involvement in substance use and poor mental health outcomes (Secades-Villa et al. 2025). Conversely, evidence also suggests that the presence of psychiatric symptoms during adolescence may increase the likelihood of initiating or sustaining cannabis use, supporting a bidirectional relationship between psychopathology and cannabis use (Gobbi et al. 2019). Young people may use cannabis to cope with symptoms such as anxiety, depression, sleep disturbances, or emotional distress, but frequent cannabis use may in turn contribute to the worsening of these symptoms and increase vulnerability to adverse mental health outcomes (Lowe et al. 2024).

Traditionally, in some epidemiological surveys and earlier population surveillance systems, particularly in contexts where cannabis was not legally regulated, cannabis use has often been operationalized as a homogeneous category, frequently labelled as “recreational” (Compton et al. 2016; Hasin et al. 2015). However, in jurisdictions where medical cannabis has been legally recognized for years, such as several states in the United States, many epidemiologic studies now distinguish between medical and non-medical use and, in some cases, assess specific motives for use (e.g., (Han et al., 2025)). By contrast, in Spain and in many countries of the European Union, national population surveys have traditionally not differentiated between medical and non-medical cannabis use, instead assessing overall prevalence of use (European Union Drugs Agency 2025). The extent to which this differentiation is incorporated therefore varies substantially across geographic settings, regulatory contexts, time periods, and data collection systems. However, motivations for use are diverse and may influence frequency, intensity, and associated health outcomes.

A growing distinction in the literature is between recreational cannabis use (RCU)**,** medical use (MCU)**,** and self-medication (SMCU)**.** RCU typically refers to consumption for pleasure, experimentation, or social reasons. MCU implies consumption within a regulated framework, with standardized preparations prescribed for specific conditions under medical supervision. SMCU, by contrast, refers to the use of cannabis to alleviate symptoms such as sleep disturbances, anxiety, or low mood without medical oversight (Cuttler et al. 2016). Nevertheless, it must be acknowledged that the boundaries between these categories are often blurred in practice. Many individuals with medical cannabis use also engage in non-medical consumption, while recreational users may self-medicate for symptoms such as social anxiety, sleep difficulties, or low mood (Turna et al. 2020). In addition, individuals who primarily use cannabis for recreational purposes may still report perceived therapeutic effects or occasional use for symptom relief (Bonn-Miller et al. 2007). Importantly, however, this conceptual differentiation remains useful for research and clinical purposes**,** yet it remains underexplored among adolescents, particularly in Spain, where medical cannabis regulation in the form of standardized preparations was enacted in October 2025. Cannabis consumption for recreational purposes is not penalized in Spain; however, its production, trafficking, and sale remain illegal. As a result, cannabis is predominantly obtained through the illicit market, most commonly via social networks such as friends and family, informal dealers, or online sources, and is primarily consumed in the form of smoked herbal cannabis (marijuana) or resin (hashish) (Observatorio Español de las Drogas y las Adicciones 2023).

Motivations for cannabis use may influence frequency, patterns of co‐use of substances, and mental health outcomes. For instance, Lin et al. (2016) found that individuals who used cannabis for medical purposes were more likely to report daily, or near-daily cannabis use and poorer overall health compared with those who reported exclusively recreational use. Similarly, findings from another study based on a nationally representative sample of U.S. adults showed that individuals who used cannabis for both medical and recreational purposes had a higher prevalence of CUD than those who used cannabis exclusively for medical or recreational purposes (Secades-Villa et al. 2026). In this study, individuals who used cannabis medically had a higher prevalence of CUD than those who used cannabis recreationally. Moreover, participants reporting both medical and recreational cannabis use endorsed poorer mental health outcomes relative to those using cannabis only for recreational or medical purposes, particularly when compared with recreational cannabis users. Providing further nuance to these patterns, a longitudinal study by Ataiants et al. (2024) identified three groups of young adult cannabis users during California’s transition to recreational legalization: “medicinal patients,” “recreational users,” and “recreational patients,” with the latter holding a medical recommendation but reporting mainly recreational motives. While medicinal users showed stable consumption patterns over time, recreational patients maintained daily or near-daily use and showed increasing problematic cannabis use (Ataiants et al. 2024). A U.S. study of young adults found that a substantial proportion of them used cannabis for various purposes and those using cannabis equally for medical and recreational purposes reported higher levels of CUD (Sridharan et al. 2024). Taken together, these findings suggest that motives for cannabis use are not trivial but central to understanding associated risks.

The Spanish regulatory context further underscores the urgency of investigating motives for cannabis use. Until recently, Spain had no standardized framework for medical cannabis. In 2022, the Spanish Congress approved a motion to regulate medical cannabis. This culminated in *Real Decreto 903/2025* (October 9, 2025), which established Spain’s first legal framework for standardized magistral preparations of cannabis-based medicines (Ministerio de Sanidad 2025). The decree limits prescribing to hospital specialists and restricts preparation and dispensing to hospital pharmacies, thereby excluding community pharmacies, primary care physicians and home cultivation. Products must be registered with the *Agencia Española de Medicamentos y Productos Sanitarios* (AEMPS) to ensure quality, traceability, and defined THC/CBD ratios. Approved indications include multiple sclerosis–related spasticity, severe refractory epilepsy, chemotherapy-induced nausea, and chronic refractory pain (European Commission 2025). Importantly, the framework is limited to standardized cannabis extracts registered by the AEMPS and does not contemplate the medical dispensing of cannabis flower products or prescriptions by general practitioners. This restrictive framework raises questions about public perceptions of “medical” cannabis and the potential risks of unsupervised self-medication among young people. Despite the high prevalence of cannabis use among Spanish youth, little is known about the relative prevalence of recreational versus medical or self-medication use in this population, or how these motives relate to mental health and other substance use. Prior Spanish studies have documented problematic cannabis use in adolescents (Villanueva-Blasco et al. 2022), individuals with tobacco co-use (Geada et al. 2018), and young adults with psychiatric comorbidity (Cuenca-Royo et al. 2013), but none have explicitly disaggregated users by motive. As a result, prevention and treatment strategies may overlook subgroups at heightened risk, such as adolescents who self-prescribe cannabis to manage sleep or mood problems, or those who combine medical and recreational motives.

The current study seeks to address these gaps by examining RCU, SMCU (the two most common forms of cannabis consumption among adolescents and young adults in Spain, given the current restricted and highly regulated medical cannabis framework) and dual cannabis use (DCU) among adolescents and young adults, and by exploring associations with substance use patterns and mental health status. Specifically, we aim to: (1) estimate the prevalence of RCU, SMCU and DCU; (2) compare groups with respect to frequency of use, age of initiation, and co-use of alcohol, tobacco, and other drugs; and (3) assess associations between type of cannabis use and indicators of mental health, including depressive and anxiety symptoms, schizotypy, difficulties in emotion regulation, sleep quality, suicidal risk and general psychological distress.

By disentangling motives for cannabis use in a young Spanish population, this study aims to provide timely evidence relevant to clinicians, policymakers, and public health stakeholders. Findings will inform regulatory implementation of medical cannabis, guide prevention strategies that address misconceptions about medical use and help identify vulnerable subgroups of adolescents and young adults for early intervention.

## Methods

### Participants and procedure

All educational institutions and university faculties listed in the public registries of three Spanish autonomous communities (Castilla & León, the Principality of Asturias, and the Valencian Community) were contacted to invite them to participate in the study. The participation rates were 28.4%, 27%, and 19%, respectively. Data were collected from 6,757 adolescents and young adults aged 16 to 30 years who were enrolled in formal education (i.e., high school, vocational training school, or university) in these three Spanish autonomous communities [Castile and León (*n* = 1,012; 15.9%), the Principality of Asturias (*n* = 3,355; 52.7%), and the Valencian Community (*n* = 1,998; 31.4%)]. Of these, 142 participants were excluded for being 31 years or older. An additional 176 were excluded because they failed attentional control checks, and 74 were identified as duplicate cases. Finally, participants (*N* = 215) who endorsed only the “relief of tension and relaxation” cannabis use motive in the SMCU and DCU groups were excluded, as this item was nearly universally endorsed and therefore provided limited discriminatory value for stratifying participants according to reinforcement-based motivational profiles. This study examined data of adolescents and young adults who had used cannabis in the past year (*N* = 1,078). Table 1 presents the sociodemographic, substance-related, and clinical characteristics of the sample.

**Table 1:** Sociodemographic, clinical and substance use characteristics among self-medicated cannabis users only (SMCU), recreational cannabis users only (RCU), and dual cannabis users (DCU) among adolescents and young adults (*N* = 1,078)

Characteristic	SMCU only (*n* = 89, 8.3%)	RCU only (*n* = 415, 38.5%)	DCU (*n* = 574, 53.2%)	Statistic	Effect size (Cohen’s *d*/Cramer’s V)
	Mean (SE)/*n* (%)	Mean (SE)/*n* (%)	Mean (SE)/*n* (%)		
*Demographics*					
Age	20.11 (0.30)	19.83 (0.12)	20.02 (0.13)	0.952^a,1^	
				0.260^b,1^	
				−1.116^c,1^	
Sex (% men)	38 (42.7%)	189 (45.5%)	294 (51.2%)	0.139^a,2^	
				1.911^b,2^	
				2.884^c,2^	
Weekly income (€)	81.20 (16.60)	56.65 (4.65)	74.30 (6.22)	1.424^a,1^	
				0.404^b,1^	
				−2.274^c,1^	
*Substance use*					
Age of onset of cannabis use	15.89 (0.22)	16.53 (0.12)	15.25 (0.10)	−2.280^a,1^	
				2.339^b,1^	
				**8.077**^c,1**^	**0.520**
Days of cannabis use (past-month)	2.42 (0.63)	0.90 (0.16)	8.77 (0.46)	2.317^a,1^	
				**−8.129**^b,1**^	**−0.606**
				**−16.127**^c,1**^	**−0.908**
CAST	2.54 (0.39)	0.48 (0.07)	5.91 (0.23)	**5.202**^a,1**^	**1.010**
				**−7.493**^b,1**^	**−0.643**
				**−22.768**^c,1**^	**−1.276**
B-MACQ	1.93 (0.37)	0.73 (0.07)	5.30 (0.20)	**3.234**^a,1*^	**0.624**
				**−8.027**^b,1**^	**−0.712**
				**−21.181**^c,1**^	**−1.188**
Past-month tobacco use	65 (73%)	217 (52.3%)	445 (77.5%)	**11.968**^a,2**^	**0.159**
				0.641^b,2^	
				**68.179**^c,2**^	**0.265**
Age of onset of tobacco use	14.73 (0.21)	15.29 (0.12)	14.25 (0.11)	−2.022^a,1^	
				1.608^b,1^	
				**6.199**^c,1**^	**0.423**
Past-month e-cigarette use	28 (31.5%)	128 (30.8%)	200 (34.8%)	0.001^a,2^	
				0.255^b,2^	
				1.563^c,2^	
HSI	1.02 (0.14)	0.51 (0.05)	1.11 (0.06)	**3.398**^a,1**^	**0.453**
				−0.583^b,1^	
				**−7.687**^c,1**^	**−0.477**
Past-month alcohol use	78 (87.6%)	366 (88.2%)	500 (87.1%)	0.001^a,2^	
				0.001^b,2^	
				0.170^c,2^	
Age of onset of alcohol use	14.28 (0.16)	14.58 (0.08)	13.84 (0.08)	−1.543^a,1^	
				2.068^b,1^	
				**6.275**^c,1**^	**0.407**
Binge drinking in the last year	68 (76.4%)	324 (78.1%)	471 (82.1%)	0.041^a,2^	
				1.268^b,2^	
				2.178^c,2^	
AUDIT	6.85 (0.49)	7.28 (0.23)	8.26 (0.25)	−0.787^a,1^	
				−2.153^b,1^	
				**−2.892**^c,1*^	**−0.180**
B-YAACQ	6.70 (0.49)	7.24 (0.22)	8.41 (0.22)	−1.030^a,1^	
				**−2.858**^b,1*^	**−0.326**
				**−3.757**^c,1**^	**−0.235**
Past-year any illicit substance use	20 (22.5%)	88 (21.2%)	230 (40.1%)	0.015^a,2^	
				**9.423**^b,2*^	**0.124**
				**38.433**^c,2**^	**0.199**
*Mental Health*					
BSI_DEP	6.19 (0.63)	5.29 (0.26)	7.97 (0.27)	1.405^a,1^	
				**−2.601**^b,1*^	**−0.275**
				**−7.043**^c,1**^	**−0.440**
BSI_ANX	5.81 (0.59)	4.54 (0.23)	6.75 (0.25)	1.992^a,1^	
				−1.421^b,1^	
				**−6.632**^c,1**^	**−0.411**
BSI_SOM	5.10 (0.54)	4.05 (0.22)	5.80 (0.23)	1.972^a,1^	
				−1.108^b,1^	
				**−5.469**^c,1**^	**−0.340**
BSI_GSI	17.10 (1.60)	13.88 (0.63)	20.53 (0.69)	2.076^a,1^	
				−1.848^b,1^	
				**−7.122**^c,1**^	**−0.441**
ESQUIZO-Q–A RD	15.89 (0.67)	14.21 (0.28)	17.03 (0.29)	**2.444**^a,1*^	**0.285**
				−1.477^b,1^	
				**−6.997**^c,1**^	**−0.439**
ESQUIZO-Q–A ANH	15.37 (0.70)	15.71 (0.35)	15.04 (0.27)	−0.415^a,1^	
				0.437^b,1^	
				1.525^c,1^	
ESQUIZO-Q–A INTD	21.33 (0.67)	20.54 (0.34)	23.02 (0.29)	0.991^a,1^	
				−2.162^b,1^	
				**−5.565**^c,1**^	**−0.359**
DERSP_ACCEPT	4.92 (0.23)	4.73 (0.10)	5.52 (0.12)	0.793^a,1^	
				−2.347^b,1^	
				**−5.066**^c,1**^	**−0.309**
DERSP_GOALS	5.97 (0.31)	5.96 (0.16)	6.79 (0.15)	0.026^a,1^	
				−2.368^b,1^	
				**−3.763**^c,1**^	**−0.237**
DERSP_IMPULSE	6.13 (0.28)	5.88 (0.12)	6.88 (0.15)	0.889^a,1^	
				−2.383^b,1^	
				**−5.280**^c,1**^	**−0.319**
PSS	0.99 (0.16)	0.78 (0.06)	1.54 (0.07)	1.332^a,1^	
				**−3.183**^b,1*^	**−0.329**
				**−7.932**^c,1**^	**−0.491**

*CAST* Cannabis Abuse Screening Test, *B-MACQ* The Brief-Marijuana Consequences Questionnaire, *HSI* The Heaviness of Smoking Index, *AUDIT* Alcohol Use Disorders Identification Test, *B-YAACQ* The Brief Young Adult Alcohol Consequences Questionnaire, *BSI_DEP* Depression dimension of the Brief Symptom Inventory-18, *BSI_ANX* Anxiety dimension of the Brief Symptom Inventory-18, *BSI_SOM* Somatization dimension of the Brief Symptom Inventory-18, *BSI_GSI* The Global Severity Index of the BSI, *ESQUIZO-Q–A RD* Reality distortion dimension of the Oviedo Schizotypy Assessment Questionnaire – Abbreviated (ESQUIZO-Q–A), *ESQUIZO-Q–A ANH* Anhedonia dimension of the ESQUIZO-Q–A, *ESQUIZO-Q–A INTD* Interpersonal disorganization dimension of the ESQUIZO-Q–A, *DERSP_ACCEPT* Non-acceptance subscale of the Difficulties in Emotion Regulation of Positive Emotions Scale, *DERSP_GOALS* Goals subscale of the Difficulties in Emotion Regulation of Positive Emotions Scale, *DERSP_IMPULSE* Impulse subscale of the Difficulties in Emotion Regulation of Positive Emotions Scale, *PSS* Paykel Suicide Scale, *SMCU* Self-medicated cannabis use, *RCU* Recreational cannabis use, *DCU* Dual Self-medicated and recreational cannabis use

^a^Comparisons between self-medicated cannabis users only and recreative users only

^b^Comparisons between self-medicated cannabis users only and combined users

^c^Comparisons between recreational cannabis users only and combined users

^1^Student’s t; ^2^Chi-squared

^*^p ≤.017

^**^p ≤.001

Assessments were administered either onsite (92%) or, when this was not possible, online (8%). Participants completed the assessment using their personal mobile phones, institutional devices (i.e., tablets or computers), or tablets provided by the researchers (Lenovo® Tab M7). Each assessment lasted approximately 45 min. The Ethics Committee of the Principality of Asturias reviewed and approved the study prior to data collection (Ref: 2022.403).

### Attentional control checks

Attentional control checks were included to identify lack of attention, random responding, or misunderstanding of the task. These items were randomly distributed throughout the assessment battery and consisted of 5 multiple-choice questions (e.g., *“In order to answer this question, select ‘sometimes’”*). Participants who failed to answer at least 3 out of 5 attentional control items correctly were excluded from the study (*n* = 176).

### Measures

Sociodemographic variables included sex at birth, age, and discretionary weekly income: amount of money available for personal expenses each week.

To classify individuals who used cannabis for self-medication purposes, respondents reporting past-year cannabis use were asked whether any of their use was for:(1) forgetting worries or problems, (2) coping with feelings of depression or anxiety, (3) relief of tension and relax, (4) falling asleep or improving sleep, or (5) relieving physical pain. These items were selected to capture motives conceptually related to the self-medication hypothesis and negative reinforcement models, in which substance use is motivated by the alleviation of psychological or physical discomfort (Ferguson et al. 2021; Spechler et al. 2020). Within this framework, self-medication use was operationalized as cannabis use intended to relieve emotional, sleep-related, or physical symptoms, encompassing motives that in the cannabis literature are often discussed within both self-medication and coping-related domains. Endorsement of these motives reflects the perceived use of cannabis for symptom relief, although it does not necessarily imply the presence of a clinically diagnosed physical or psychological condition. To classify individuals who used cannabis for recreational purposes, respondents reporting past-year cannabis use were asked whether any of their use was for: (1) fitting in with a group, (2) enhancing thinking or creativity, (3) enjoying a party, or (4) liking the feeling. These items were selected to capture motives corresponding to the social, enhancement, conformity, and expansion domains described in established motivational models of cannabis use, particularly the Marijuana Motives Measure (MMM) and related frameworks (Simons et al. 1998; Lee et al. 2007). In these models, conformity motives (e.g., fitting in with a group) are conceptualized as externally driven social reinforcement motives, whereas coping and symptom-relief motives are conceptualized as internally driven negative reinforcement motives. Participants who endorsed at least one of the five self-medication items and none of the recreational items were classified as self-medication users. Those who endorsed none of the self-medication items and at least one of the recreational items were classified as recreational users. Participants who endorsed at least one self-medication item and at least one recreational item were classified as dual (self-medication and recreational) users.

Self-report questions on substance use assessed: past-year prevalence of illegal substances; past-year prevalence of binge drinking, past-month prevalence of tobacco, e-cigarettes, alcohol, cannabis, and; past-month frequency of cannabis use (i.e., number of days); and age of onset of tobacco, alcohol, cannabis.

Participants who reported past-month tobacco use completed the Heaviness of Smoking Index (HSI) (Heatherton et al. 1989), a two-item self-report questionnaire assessing nicotine dependence. The HSI evaluates quantity of tobacco use (*“How many cigarettes per day do you smoke?”*) and level of physical dependence (*“How soon after you wake up do you smoke your first cigarette?”*). Scores range from 0 to 6, with scores of 4 or higher indicating tobacco dependence. The HSI has demonstrated validity in samples of young adults (Meneses-Gaya et al. 2009). The reliability of the HSI in the present sample was low (r_*s*_ = 0.401), arguably because it consists of only two items, thus underestimating its reliability (Tavakol and Dennick 2011).

All participants completed the Cannabis Abuse Screening Test (CAST) (Legleye et al. 2011). This six-item questionnaire uses a five-point Likert scale (0 = never; 4 = very often) to produce a total score ranging from 0 to 24, indicative of the risk of Cannabis Use Disorder (CUD) in the past year. A score of 7 or higher indicates moderate dependence, while a score of 12 or higher indicates severe dependence. The CAST has shown excellent internal consistency among young people (Cuenca-Royo et al. 2012). The internal consistency in the current sample was excellent (α = 0.865). An example of a CAST item is the following: “Have you ever tried to cut down your cannabis use?”.

The negative consequences of cannabis use were assessed using the 21-item Brief-Marijuana Consequences Questionnaire (B-MACQ). Each item evaluates a specific cannabis-related consequence experienced in the past month, with higher total scores indicating greater severity of adverse outcomes. The B-MACQ has demonstrated good reliability and validity in prior research and is widely used to capture the functional and psychosocial impacts of cannabis use in youth populations (Bravo et al. 2019). Internal consistency in the current sample was excellent (α = 0.906). An example of a B-MACQ item is the following: “I have had problems with memory because of my cannabis use.”

The Alcohol Use Disorders Identification Test (AUDIT) (Saunders et al. 1993) was used to assess past-year risk of Alcohol Use Disorder (AUD). The AUDIT is a 10-item Likert-type questionnaire; cut-off scores for high-risk drinking are 8 for men and 6 for women (Carretero et al. 2016). A score of 13 or higher indicates possible alcohol dependence. Research supports the use of the AUDIT as a screening tool for the spectrum of alcohol use disorders across diverse settings and populations (Reinert and Allen 2007). An example of an AUDIT item is the following: “How often do you have a drink containing alcohol?”.

The Brief Young Adult Alcohol Consequences Questionnaire (B-YAACQ) (Kahler et al. 2005) was used to measure past-year negative consequences of alcohol consumption. The B-YAACQ is a 24-item questionnaire with dichotomous responses, yielding a total score ranging from 0 to 24 that reflects the number of negative alcohol-related consequences. Higher scores indicate more severe consequences of alcohol use. An example of a B-YAACQ item is the following: “I have had a hangover the morning after I had been drinking.”. Both the AUDIT and the B-YAACQ have demonstrated strong reliability among young adult populations (Carretero et al. 2016; Espejo-Tort 2020). In the current sample, both questionnaires showed adequate reliability (AUDITα = 0.776; B-YAACQα = 0.867).

The Brief Symptom Inventory-18 (BSI-18) (Derogatis 2001, 2013) was used to measure risk of emotional disorders and related symptomatology (i.e., depression, anxiety, and somatization) during the week prior to assessment. This self-administered questionnaire includes 18 items rated on a five-point Likert scale (0 = not at all; 4 = extremely). Scores can be summed to yield the Global Severity Index (GSI), a measure of overall psychological distress. Higher GSI scores indicate greater risk of emotional disorder. The BSI-18 has demonstrated good reliability in samples including young adults (Iza-Fernández et al. 2025). Excellent reliability was observed in the current study sample for both the GSI (α = 0.940), and subscales (depression: α = 0.888; anxiety: α = 0.854; somatization α = 0.852).An example of a BSI-18 item is the following: “During the past 7 days, how much were you distressed by feeling no interest in things?”.

Schizotypal traits were assessed with the Oviedo Schizotypy Assessment Questionnaire–Abbreviated (ESQUIZO-Q–A) (Fonseca-Pedrero et al. 2010). This 23-item questionnaire uses a five-point Likert scale (1 = totally disagree; 5 = totally agree) and includes three subscales: *Reality Distortion (RD)*, consisting of 6 items (e.g., magical thinking, unusual perceptual experiences, paranoid ideation); *Anhedonia (ANH)*, with 7 reverse-scored items (e.g., physical and social anhedonia); and *Interpersonal Disorganization (INTD)*, with 10 items (e.g., odd thinking and speech, ideas of reference, odd behavior, lack of close friends, excessive social anxiety). Subscale scores are obtained by summing the relevant items, with higher scores indicating greater likelihood of schizotypal traits. The ESQUIZO-Q–A has shown evidence of reliability in adolescent general population samples (Fonseca-Pedrero et al. 2012). The internal reliability in this study sample was good, including the total score (α = 0.893), and its dimensions (reality distortion α = 0.830; anhedonia α = 0.863; interpersonal disorganization α = 0.816). An example of a ESQUIZO-Q–A item is the following: “I feel good when I see my friends and family happy”.

The Difficulties in Emotion Regulation of Positive Emotions Scale (DERS-P) (Weiss et al. 2015) comprises 13 items, with a 5-point Likert format ranging from 1 (almost never) to 5 (almost always). The total score is calculated by the sum of the individual items, where a higher total score indicates greater difficulties in regulating positive emotions. It allows to compute three dimensions: Acceptance (a tendency to react critically or negatively toward positive emotional experiences), Goals (challenges in maintaining goal-directed behavior in the presence of positive emotions), and Impulse (difficulties in regulating impulsive behaviors during positive emotional states). The DERS-P has demonstrated reliability in young adult populations (Weiss et al. 2019). In the current sample, reliability was excellent for both the total score (α = 0.821) and subscales (accept: α = 0.869; goals: α = 0.910; impulse: α = 0.902). An example of a DERS-P item is the following: “When I'm happy, I feel ashamed of myself for feeling that way”.

Finally, suicidal risk in the previous 12 months was assessed with the Paykel Suicide Scale (PSS) (Paykel et al. 1974), a brief five-item questionnaire that measures risk of suicidal behaviors. Items range from lower-risk behaviors (e.g., desire to die) to higher-risk behaviors (e.g., suicidal ideation and attempts). The PSS is unidimensional, with higher scores indicating greater suicidal risk. This scale has demonstrated excellent reliability among young populations (Fonseca-Pedrero et al. 2020) and in the current sample (α = 0.82). An example of a PSS item is the following: “During the past year, have you felt that life was not worth living?".

### Analyses

Descriptive statistics (frequencies and percentages for categorical variables and mean and standard deviation for continuous variables) were carried out to characterize the study’s sample. A set of bivariate comparisons of sociodemographic, substance use and clinical characteristics were conducted across those with SMCU only, RCU only, and DCU utilizing log likelihood chi-square tests (after Yate’s continuity correction) for categorical variables and independent *t*-test for continuous variables. To control for inflated Type I error due to multiple pairwise comparisons, Bonferroni adjustments for multiple testing were conducted by dividing the nominal alpha level (0.05) by the number of comparisons conducted for each dependent variable (i.e., 3), resulting in a more stringent significance threshold of ≤ 0.017. To identify variables independently associated with user-group classification (i.e., SMCU and DCU vs. reference category = RCU), multinomial logistic regression models were estimated, given the mutually exclusive nature of the three cannabis use categories. The independent variables (predictors) were selected a priori based on theoretical considerations and previous empirical literature on cannabis use, substance use, and mental health among adolescents and young adults (Secades-Villa et al. 2025; Stiles-Shields et al. 2023; Jeffers et al. 2021; Crane et al. 2015; Albertella et al. 2017). The predictors included age, sex, discretionary weekly income, age of onset of cannabis, tobacco, and alcohol use, number of days of cannabis, tobacco, and alcohol use in the past 30 days, past-year illegal drug use (yes/no), cannabis-related problems, nicotine dependence, AUD risk, depression, anxiety, somatization, anhedonia, reality distortion, positive emotion dysregulation, and suicidal risk. Multicollinearity assessment (i.e., tolerance and variance inflation factor [VI]) was conducted to identify whether two or more of the independent predictors included in the regression model were highly correlated with each other. Results are reported as odds ratios (ORs) with 95% confidence intervals (CIs). Tolerance values below 0.20 and VIF values above 5 were considered indicative of problematic multicollinearity (Kim 2019).

All analyses were conducted using IBM SPSS statistics, version 27.0.1.0 (Corp 2020).

### Transparency and openness

Materials and analysis code for this study are available by emailing the corresponding author.

## Results

Of the 1,078 adolescents and young adults with any past-year cannabis use, 8.3% (*n* = 89) reported self-medicated only use, 38.5% (*n* = 415) reported recreational only use, and 53.2% (*n* = 574) reported combined self-medicated and recreational use. The most frequently reported reasons for using cannabis as self-medication were, in descending order: relief of tension and relax (90.5%), escapism or forgetting worries and problems (65.2%), improving or inducing sleep (65%), coping with depressive or anxious feelings (59.9%), and relieving physical pain (35.1%).

The frequencies of reasons for recreational use were liking the feeling (70.7%), enhancing thinking or creativity (26.3%), enjoying a party (45.9%), and fitting in with a group (11.1%).

### Bivariate analyses

Table 1 presents the demographic, substance use, and mental health characteristics of the three groups (SMCU, RCU, and DCU), along with their bivariate comparisons. Significant between-group differences were observed in 21 characteristics between the RCU and DCU groups (see statistics marked with the c superscript), and in seven characteristics between the SMCU and DCU groups (marked with b superscript). Overall, the DCU group exhibited a more severe profile in terms of both substance use and mental health compared with the RCU and SMCU groups. The SMCU and RCU groups were more similar (see statistics marked with a superscript), and differed in four substance use variables, with SMCU users displaying a more severe substance use profile than RCU users. Additionally, SMCU users scored higher than recreational users on the Reality Distortion subscale of the ESQUIZO-Q–A.

### Multinomial logistic regression

Table 2 displays the results of the multinomial logistic regression with RCU as the reference category. Supplementary Table 1 presents the results of the same multinominal logistic regression model using age as a continuous variable. Statistically significant predictors of SMCU were as follows: higher weekly income (OR = 1.002, 95% CI = 1.000, 1.004, *p* = 0.015), higher cannabis-related problems (OR = 1.599, 95% CI = 1.372, 1.865, *p* < 0.001), greater nicotine dependence (OR = 1.275, 95% CI = 1.026, 1.585, *p* = 0.028). Variables predicting DCU included more frequent cannabis use within the past month (OR = 1.076, 95% CI = 1.008, 1.149, *p* = 0.028), higher cannabis-related problems (OR = 1.870, 95% CI = 1.633, 2.141, *p* < 0.001), earlier age of alcohol use initiation (OR = 0.846, 95% CI = 0.739, 0.968, *p* = 0.015).

**Table 2:** Predictors of self-medicated and dual cannabis use vs. recreational cannabis us

Variables	*B*	SE	Wald’s Statistic	*p* value	Odds ratio (Exp (B))	95% C.I. for Odds Ratio		Tolerance	VIF
Self-medicated cannabis users (SMCU)									
Sex (ref. women)	-.168	.290	.337	.561	0.845	.479	1.491	.821	1.218
Age (ref. underage)	.047	.386	.015	.904	1.048	.492	2.233	.813	1.230
Weekly income	.002	.001	5.880	**.015**	1.002	1.000	1.004	.937	1.067
Age of onset of cannabis use	-.027	.077	.121	.728	.974	.837	1.132	.510	1.962
Days of cannabis use (past-month)	.020	.040	.254	.614	1.021	.943	1.104	.527	1.898
CAST	.470	.078	35.900	** <.001**	1.599	1.372	1.865	.495	2.020
Age of onset of tobacco use	.053	.079	.443	.506	1.054	.903	1.230	.482	2.074
Days of tobacco use (past-month)	.021	.012	2.858	.091	1.021	.997	1.046	.644	1.554
HSI	.243	.111	4.822	**.028**	1.275	1.026	1.585	.750	1.334
Age of onset of alcohol use	-.143	.099	2.096	.148	.867	.715	1.052	.653	1.531
Days of alcohol use (past-month)	-.013	.029	.209	.648	.987	.933	1.044	.691	1.446
AUDIT	-.039	.033	1.382	.240	.962	.901	1.026	.663	1.509
Past-year any illicit substance use (ref. no)	-.637	.338	3.555	.059	.529	.273	1.025	.796	1.257
BSI_DEP	-.011	.041	.070	.791	.989	.913	1.072	.308	3.246
BSI_ANX	.063	.049	1.642	.200	1.065	.967	1.172	.242	4.125
BSI_SOM	-.019	.044	.182	.669	.982	.901	1.069	.335	2.985
ESQUIZO-Q–A ANH	-.019	.024	.615	.433	.981	.936	1.029	.637	1.570
ESQUIZO-Q–A RD	.022	.027	.668	.414	1.022	.970	1.077	.556	1.798
ESQUIZO-Q–A INTD	-.013	.029	.189	.664	.987	.932	1.046	.400	2.497
DERSP_ACCEPT	.002	.077	.001	.975	1.002	.861	1.167	.565	1.770
DERSP_GOALS	-.080	.058	1.925	.165	.923	.824	1.034	.511	1.958
DERSP_IMPULSE	.027	.069	.151	.697	1.027	.897	1.176	.465	2.149
PSS	.088	.125	.499	.480	1.092	.855	1.394	.501	1.997
Dual cannabis users (DCU)									
Sex (ref. women)	.157	.197	.633	.426	1.170	.795	1.720	.821	1.218
Age (ref. underage)	-.199	.262	.575	.448	.820	.491	1.370	.813	1.230
Weekly income	.001	.001	1.585	.208	1.001	.999	1.003	.937	1.067
Age of onset of cannabis use	-.017	.050	.122	.727	.983	.891	1.084	.510	1.962
Days of cannabis use (past-month)	.073	.033	4.822	**.028**	1.076	1.008	1.149	.527	1.898
CAST	.626	.069	81.947	** <.001**	1.870	1.633	2.141	.495	2.020
Age of onset of tobacco use	.045	.052	.740	.390	1.046	.945	1.158	.482	2.074
Days of tobacco use (past-month)	.003	.009	.140	.708	1.003	.986	1.021	.644	1.554
HSI	.128	.083	.2.379	.123	1.137	.966	1.338	.750	1.334
Age of onset of alcohol use	-.167	.069	5.907	**.015**	.846	.739	.968	.653	1.531
Days of alcohol use (past-month)	.006	.020	.097	.755	1.006	.968	1.046	.691	1.446
AUDIT	-.030	.022	1.846	.174	.970	.929	1.013	.663	1.509
Past-year any illicit substance use (ref. no)	-.372	.230	2.629	.105	.689	.439	1.081	.796	1.257
BSI_DEP	.044	.027	2.670	.102	1.045	.991	1.101	.308	3.246
BSI_ANX	.002	.033	.006	.940	1.002	.939	1.070	.242	4.125
BSI_SOM	-.018	.030	.379	.538	.982	.926	1.041	.335	2.985
ESQUIZO-Q–A ANH	-.012	.017	.521	.471	.988	.955	1.021	.637	1.570
ESQUIZO-Q–A RD	-.002	.019	.011	.915	.998	.962	1.035	.556	1.798
ESQUIZO-Q–A INTD	.022	.020	1.124	.289	1.022	.982	1.064	.400	2.497
DERSP_ACCEPT	.034	.048	.491	.484	1.034	.941	1.137	.565	1.770
DERSP_GOALS	-.006	.034	.029	.866	.994	.929	1.064	.511	1.958
DERSP_IMPULSE	.009	.044	.045	.833	1.009	.926	1.101	.465	2.149
PSS	.160	.084	3.624	.057	1.173	.995	1.382	.501	1.997

Reference group: recreational users

*CAST* Cannabis Abuse Screening Test, *HSI* the Heaviness of Smoking Index, *AUDIT* Alcohol Use Disorders Identification Test, *BSI_DEP* Depression dimension of the Brief Symptom Inventory-18, *BSI_ANX* Anxiety dimension of the Brief Symptom Inventory-18, *BSI_SOM* Somatization dimension of the Brief Symptom Inventory-18, *ESQUIZO-Q–A ANH* Anhedonia dimension of the Oviedo Schizotypy Assessment Questionnaire – Abbreviated (ESQUIZO-Q–A), *ESQUIZO-Q–A RD* Reality distortion dimension of the ESQUIZO-Q–A, *ESQUIZO-Q–A INTD* Interpersonal disorganization dimension of the ESQUIZO-Q–A, *DERSP_ACCEPT* Non-acceptance subscale of the Difficulties in Emotion Regulation of Positive Emotions Scale, *DERSP_GOALS* Goals subscale of the Difficulties in Emotion Regulation of Positive Emotions Scale, *DERSP_IMPULSE* Impulse subscale of the Difficulties in Emotion Regulation of Positive Emotions Scale, *PSS* Paykel Suicide Scale

The multinomial regression model showed a significantly better fit than the null model (χ^2^ = 528.199, df = 46, *p* <.001) and lower − 2 log likelihood (1219.168) and AIC (1315.168) values, indicating improved fit. OR = odds ratio; CI = confidence interval

## Discussion and conclusions

This study examined differences among three groups of young cannabis users (recreational, self-medicated, and dual) in sociodemographic characteristics, substance use patterns, and mental health indicators. Our findings indicate that most of the adolescents and young adults (53.2%) reported DCU, with smaller proportions engaging exclusively in RCU and less in SMCU. Importantly, dual users displayed the most severe overall pattern, characterized by heavier cannabis consumption, greater risk of CUD, higher polysubstance involvement, and poorer mental health. SMCU showed intermediate levels of severity, with higher cannabis and nicotine dependence but similar mental health scores compared with recreational users. Recreational users exhibited the lowest risk across both substance use and psychological domains.

The predominance of dual users suggests that cannabis use in young people often serves multiple functions rather than a single, discrete motive. This aligns with recent research indicating that recreational and self-medicative motives frequently co-occur (Turna et al. 2020; Wallez et al. 2024; Rotermann and Pagé 2018). This pattern may reflect the complex interplay between recreational enjoyment and attempts to self-manage psychological or physical symptoms, highlighting that motives for use are not mutually exclusive. The relatively smaller proportions of exclusive self-medicated or recreational users suggest that targeting interventions based solely on a single motive may overlook a substantial segment of high-risk youth who use cannabis for multiple purposes.

Consistent with previous studies (Green et al. 2003; Hyman and Sinha 2009), our findings show that tension relief and relaxation was the most frequent reason for self-medicated cannabis use (55.7%), followed by escapism, sleep improvement, and emotional relief. This pattern suggests that most young people use cannabis to manage everyday stress rather than to treat or relieve specific physical pain. This result diverges from prior research reporting the use of non-prescribed cannabis to manage physician-diagnosed medical conditions (Strada et al. 2025). This difference may reflect variations in sample characteristics, as only about 20% of participants in Strada et al. were under 30 years of age, whereas the present study focuses on a younger population (Strada et al. 2025). Age-related differences in health profiles and coping strategies may influence motivations for self-medicated cannabis use, with younger individuals being more likely to report stress-related and emotional reasons rather than the management of physician-diagnosed medical conditions. Such coping motives may reinforce habitual use and increase the risk of dependence (Johnson et al. 2010; Glodosky and Cuttler 2020), highlighting the need for preventive interventions that promote healthier strategies for stress and sleep management.

Consistent with previous research (Cuttler et al. 2016; Lin et al. 2016; Amiet et al. 2020), dual users exhibited the most severe pattern of cannabis use, with higher frequency of use, and more cannabis-related consequences, as reflected by elevated scores on the CAST and the B-MACQ compared to the remaining groups. Higher frequency of cannabis use in the past-month, greater cannabis-related problems, and earlier age of alcohol onset were significant correlates of belonging to the DCU group, suggesting that engaging in cannabis use for both recreational and self-medication may synergistically reinforce both frequency and dependence risk. The coexistence of enhancement and coping motives may reinforce consumption through both positive and negative reinforcement mechanisms (Bonn-Miller et al. 2007; Bresin and Mekawi 2019). Additionally, dual users exhibited higher AUDIT and B-YAACQ scores than RCU**,** indicating greater alcohol-related consequences, which may amplify the overall burden of risk through polysubstance interactions.

Dual users also exhibited higher levels of depressive and anxiety symptoms, schizotypal traits, and suicidal risk, reflecting a more clinically impaired profile and highlighting the coexistence of psychological vulnerability and heavy cannabis involvement (Gobbi et al. 2019; Maffre Maviel et al. 2025). Similar findings have been reported in North American samples, where individuals endorsing both recreational and self-medication motives exhibit greater psychiatric vulnerability and heavier cannabis use (Turna et al. 2020; Ataiants et al. 2024). These findings are also consistent with previous studies showing that self-medicated cannabis use is primarily motivated by the management of mental health problems, including anxiety, sleep disorders or depression (Asselin et al. 2022; Sexton et al. 2016; Wallis et al. 2022). Nevertheless, the causal relationship is likely bidirectional: young people with underlying emotional or psychotic-like symptoms may be more likely to use cannabis, while heavy use may, in turn, exacerbate emotional dysregulation and cognitive-perceptual disturbances (Scott et al. 2018; Muñoz-Galán et al. 2023).

In contrast, comparisons between self-medicated and recreational users revealed fewer differences. Nonetheless, self-medicated users demonstrated greater CUD risk and higher nicotine dependence than recreational ones, consistent with prior findings associating coping motives with higher substance involvement and lower self-regulatory control (Lin et al. 2016). Despite reporting self-medication motives, these users did not differ significantly from recreational users in depressive, anxious, or somatic symptoms, indicating that their use may reflect perceived rather than clinically verified distress. However, they did show higher scores on the Reality Distortion subscale of the ESQUIZO-Q–A, suggesting greater experiences related to perceptual and cognitive distortions. Overall, these findings may indicate that self-medication motives in this group are more closely linked to subjective experiences of distress rather than to clinically elevated affective symptomatology. This interpretation aligns with prior work suggesting that young users often self-identify as self-medicating without formal diagnosis or effective symptom reduction, and that self-medication is based on perceived rather than clinically verified distress.

Taken together, these results highlight the importance of assessing cannabis use motives in adolescents and young adults, as self-medicated and dual users appear to be at higher risk for negative outcomes, including substance-related problems, co-occurring mental health conditions, and suicide risk, particularly those combining recreational and self-medicated motives. Early identification and targeted interventions, potentially integrating mental health support and substance use prevention, may help mitigate these risks. Furthermore, the strong association between tobacco and cannabis use in the self-medicated group suggests that interventions addressing co-use may be particularly important in this subgroup.

This study contributes novel data from a large and diverse sample of Spanish adolescents and young adults, representing one of the first motive-based analyses of cannabis use in a European context where cannabis for recreational purposes remains unregulated. The use of validated instruments across substance use, mental health, and emotion regulation domains enhances the robustness of the findings. However, several limitations must be acknowledged. The cross-sectional design precludes causal inferences, and the reliance on self-report measures introduces potential recall and social desirability biases. The operationalization of “self-medication” was based on perceived motives rather than clinical evaluation, potentially overestimating its prevalence. Future research should further refine the operationalization of self-medication motives by distinguishing between cannabis use intended to alleviate physician-diagnosed medical conditions and use aimed at self-perceived but undiagnosed health problems. Additionally, the self-medication versus recreational classification was adapted for this study to apply a theory-driven, inclusive approach, and psychometric validation in this sample was not conducted. Finally, the sample was limited to students, restricting generalizability to non-student or high-risk populations. Future research should employ longitudinal designs and structured psychiatric assessments to clarify causal pathways and examine transitions between motive-based user groups over time.

Despite these limitations, this study provides evidence of distinct risk profiles among adolescent cannabis users based on their reported motives for use and highlights the importance of distinguishing between recreational, self-medicated, and dual motives when assessing risk. Dual users (those who consume for both recreational and self-medicative purposes) emerged as a particularly vulnerable subgroup characterized by heavier consumption, greater cannabis-related consequences, and poorer mental health. Prevention and early intervention programs should incorporate motive-based screening approaches and address the heterogeneity of reasons for use, while also considering the potential overlap between recreational use and attempts to self-manage psychological or emotional difficulties. In addition, these findings underline the importance of clarifying users’ perceptions of cannabis as a coping strategy in relation to unregulated use and mental health symptoms. As Spain moves toward implementing a medical cannabis framework (Ministerio de Sanidad 2025), public health initiatives should emphasize the distinction between regulated medical use and self-medication, which may perpetuate cannabis misuse and psychiatric risk among youth. Clear communication about risks associated with unregulated self-medication among youth will be essential to prevent misuse, polysubstance involvement, and worsening mental health outcomes in the context of Spain’s transitional regulatory context.

## Supplementary Information

Supplementary Material 1.

## Data Availability

The datasets used and/or analysed during the current study are available from the corresponding author on reasonable request.
