# Supplementary material for: Recreational versus self-medicated cannabis use among adolescents and young adults in Spain: associations with substance use and mental health
Source: J Cannabis Res. 2026 Aug 4;8:117. doi: 10.1186/s42238-026-00480-2 (PMC13721289; doi:10.1186/s42238-026-00480-2)
Supplement: Supplementary file 1 [file 42238_2026_480_MOESM1_ESM.docx]

| **Supplementary Table 1.**  *Predictors of self-medicated and dual cannabis use vs. recreational cannabis use using age as a continuous variable* | | | | | | | |  |  |
| --- | --- | --- | --- | --- | --- | --- | --- | --- | --- |
| Variables | *B* | SE | Wald’s Statistic | *p* value | Odds ratio (Exp (B)) | 95% C.I. for  Odds Ratio | | Tolerance | VIF |
| **Self-medicated cannabis users (SMCU)** | | | | | | | |  |  |
| Sex (ref. women) | -.157 | .289 | .296 | .587 | .854 | .485 | 1.506 | .821 | 1.218 |
| Age | .033 | .058 | .333 | .564 | 1.034 | .923 | 1.158 | .756 | 1.323 |
| Weekly income | .002 | .001 | 4.941 | **.026** | 1.002 | 1.000 | 1.004 | .910 | 1.099 |
| Age of onset of cannabis use | -.034 | .078 | .189 | .664 | .967 | .830 | 1.126 | .499 | 2.004 |
| Days of cannabis use (past-month) | .019 | .041 | .226 | .634 | 1.020 | .941 | 1.104 | .514 | 1.946 |
| CAST | .468 | .078 | 35.561 | **<.001** | 1.596 | 1.369 | 1.862 | .495 | 2.021 |
| Age of onset of tobacco use | .048 | .080 | .358 | .549 | 1.049 | .897 | 1.226 | .483 | 2.071 |
| Days of tobacco use (past-month) | .020 | .012 | 2.600 | .107 | 1.020 | .996 | 1.045 | .643 | 1.555 |
| HSI | .251 | .112 | 5.055 | **.025** | 1.285 | 1.033 | 1.599 | .744 | 1.345 |
| Age of onset of alcohol use | -.145 | .097 | 2.234 | .135 | .865 | .715 | 1.046 | .661 | 1.513 |
| Days of alcohol use (past-month) | -.015 | .029 | .271 | .603 | .985 | .931 | 1.042 | .701 | 1.428 |
| AUDIT | -.038 | .033 | 1.297 | .255 | .963 | .902 | 1.028 | .661 | 1.513 |
| Past-year any illicit substance use (ref. no) | -.654 | .339 | 3.728 | .054 | .520 | .268 | 1.010 | .790 | 1.266 |
| BSI_DEP | -.011 | .041 | .073 | .787 | .989 | .913 | 1.072 | .311 | 3.219 |
| BSI_ANX | .062 | .049 | 1.581 | .209 | 1.064 | .966 | 1.172 | .242 | 4.126 |
| BSI_SOM | -.018 | .044 | .162 | .687 | .987 | .902 | 1.070 | .335 | 2.981 |
| ESQUIZO-Q-A ANH | -.018 | .024 | .523 | .470 | .983 | .937 | 1.031 | .631 | 1.584 |
| ESQUIZO-Q-A RD | .024 | .027 | .804 | .370 | 1.024 | .972 | 1.080 | .557 | 1.794 |
| ESQUIZO-Q-A INTD | -.012 | .030 | .177 | .674 | .988 | .932 | 1.046 | .402 | 2.489 |
| DERSP_ACCEPT | .001 | .077 | <.001 | .988 | 1.001 | .860 | 1.165 | .565 | 1.771 |
| DERSP_GOALS | -.077 | .058 | 1.777 | .183 | .926 | .826 | 1.037 | .511 | 1.958 |
| DERSP_IMPULSE | .023 | .069 | .113 | .737 | 1.023 | .894 | 1.172 | .466 | 2.147 |
| PSS | .092 | .124 | .547 | .459 | 1.096 | .859 | 1.399 | .501 | 1.996 |
| **Dual cannabis users (DCU)** | | | | | | | |  |  |
| Sex (ref. women) | .176 | .197 | .804 | .370 | 1.193 | .811 | 1.753 | .821 | 1.218 |
| Age (ref. underage) | .022 | .042 | .283 | .595 | 1.023 | .942 | 1.110 | .756 | 1.323 |
| Weekly income | .001 | .001 | .937 | .333 | 1.001 | .999 | 1.003 | .910 | 1.099 |
| Age of onset of cannabis use | -.029 | .050 | .322 | .571 | .972 | .880 | 1.073 | .499 | 2.004 |
| Days of cannabis use (past-month) | .072 | .034 | 4.553 | **.033** | 1.075 | 1.006 | 1.149 | .514 | 1.946 |
| CAST | .624 | .069 | 81.222 | **<.001** | 1.866 | 1.629 | 2.137 | .495 | 2.021 |
| Age of onset of tobacco use | .036 | .052 | .482 | .488 | 1.037 | .936 | 1.149 | .483 | 2.071 |
| Days of tobacco use (past-month) | .003 | .009 | .080 | .777 | 1.003 | .985 | 1.020 | .643 | 1.555 |
| HSI | .136 | .084 | 2.643 | .104 | 1.146 | .972 | 1.349 | .744 | 1.345 |
| Age of onset of alcohol use | -.177 | .068 | 6.784 | **.009** | .838 | .734 | .957 | .661 | 1.513 |
| Days of alcohol use (past-month) | .003 | .020 | .018 | .894 | 1.003 | .965 | 1.042 | .701 | 1.428 |
| AUDIT | -.030 | .022 | 1.817 | .178 | .971 | .929 | 1.014 | .661 | 1.513 |
| Past-year any illicit substance use (ref. no) | -.398 | .230 | 2.985 | .084 | .672 | .428 | 1.055 | .790 | 1.266 |
| BSI_DEP | .046 | .027 | 2.922 | .087 | 1.047 | .993 | 1.103 | .311 | 3.219 |
| BSI_ANX | .001 | .033 | .002 | .966 | 1.001 | .938 | 1.069 | .242 | 4.126 |
| BSI_SOM | -.019 | .030 | .401 | .527 | .981 | .926 | 1.040 | .335 | 2.981 |
| ESQUIZO-Q-A ANH | -.010 | .017 | .357 | .550 | .990 | .957 | 1.024 | .631 | 1.584 |
| ESQUIZO-Q-A RD | .002 | .019 | .015 | .902 | 1.002 | .966 | 1.040 | .557 | 1.794 |
| ESQUIZO-Q-A INTD | .021 | .020 | 1.019 | .313 | 1.021 | .981 | 1.063 | .402 | 2.489 |
| DERSP_ACCEPT | .031 | .048 | .402 | .526 | 1.031 | .938 | 1.133 | .565 | 1.771 |
| DERSP_GOALS | -.002 | .034 | .004 | .952 | .998 | .933 | 1.068 | .511 | 1.958 |
| DERSP_IMPULSE | .004 | .044 | .010 | .922 | 1.004 | .921 | 1.095 | .466 | 2.147 |
| PSS | .162 | .084 | 3.727 | .054 | 1.176 | .998 | 1.386 | .501 | 1.996 |
| *Note*. Reference group: recreational users. CAST = Cannabis Abuse Screening Test; HSI = the Heaviness of Smoking Index; AUDIT = Alcohol Use Disorders Identification Test; BSI_DEP = depression dimension of the Brief Symptom Inventory-18; BSI_ANX = anxiety dimension of the Brief Symptom Inventory-18; BSI_SOM = somatization dimension of the Brief Symptom Inventory-18; ESQUIZO-Q-A ANH = anhedonia dimension of the Oviedo Schizotypy Assessment Questionnaire – Abbreviated (ESQUIZO-Q-A); ESQUIZO-Q-A RD = reality distortion dimension of the ESQUIZO-Q-A; ESQUIZO-Q-A INTD = interpersonal disorganization dimension of the ESQUIZO-Q-A; DERSP_ACCEPT = Non-acceptance subscale of the Difficulties in Emotion Regulation of Positive Emotions Scale; DERSP_GOALS = Goals subscale of the Difficulties in Emotion Regulation of Positive Emotions Scale; DERSP_IMPULSE = Impulse subscale of the Difficulties in Emotion Regulation of Positive Emotions Scale; PSS = Paykel Suicide Scale.  The multinomial regression model showed a significantly better fit than the null model (χ² = 527.809, df = 46, p < .001) and lower −2 log likelihood (1219.558) and AIC (1315.558) values, indicating improved fit. OR = odds ratio; CI = confidence interval. | | | | | | | | | |
